# Human biomonitoring from an environmental justice perspective: supporting study participation of women of Turkish and Moroccan descent

**DOI:** 10.1186/s12940-017-0260-2

**Published:** 2017-05-19

**Authors:** Bert Morrens, Elly Den Hond, Greet Schoeters, Dries Coertjens, Ann Colles, Tim S. Nawrot, Willy Baeyens, Stefaan De Henauw, Vera Nelen, Ilse Loots

**Affiliations:** 1 Department of Sociology, Faculty of Social Sciences, University of Antwerp, Sint-Jacobstraat 2, 2000 Antwerp, Belgium; 2 Provincial Institute for Hygiene, Antwerp, Belgium; 3 Environmental Health and Risk, VITO, Mol, Belgium; 4 Department of Biomedical Sciences, University of Antwerp, Antwerp, Belgium; 5 Environmental Medicine, University of Southern Denmark, Odense, Denmark; 6 Centre for Environmental Sciences, Hasselt University, Hasselt, Belgium; 7 Department of Public Health & Primary Care, Leuven University, Leuven, Belgium; 8 Analytical, Environmental and Geo-Chemistry (AMGC), Vrije Universiteit Brussel, Brussels, Belgium; 9 Department of Public Health, University of Ghent, Ghent, Belgium

**Keywords:** Human biomonitoring, Recruitment, Participation, Barriers, Socially disadvantaged groups, Ethnic minorities, Environmental justice, Study design

## Abstract

**Background:**

Environmental justice research shows how socially disadvantaged groups are more exposed and more vulnerable to environmental pollution. At the same time, these groups are less represented and, thus, less visible in biomedical studies. This socioeconomic participation bias is a form of environmental injustice within research practice itself.

**Methods:**

We designed, implemented and evaluated a targeted recruitment strategy to enhance the participation of socially disadvantaged pregnant women in a human biomonitoring study in Belgium. We focused on women of Turkish and Moroccan descent and developed a setup using personal buddies that enabled information transfer about study conditions in the pre-parturition period as well as support and follow-up with questionnaires in the post-parturition period.

**Results:**

We identified four barriers to the participation of women with a vulnerable social and ethnic background which were related to psychosocial and situational factors. Lack of trust in researchers and no perceived study benefits were important personal barriers; the complex study design and difficult self-administered questionnaires were equally significant barriers.

**Conclusion:**

By investing in direct, person-to-person contact with trusted buddies and supported by practical advice about cultural and linguistic sensitivity, it was possible to increase study participation of socially disadvantaged people. Above all, this required openness and flexibility in the mind-set of researchers so that study design and procedures could be better grounded in the experiences and circumstances of underprivileged groups.

## Background

Environmental justice research describes the disproportionate effects of environmental pollution on socially disadvantaged populations [1, 2]. People living in poverty, having a low socioeconomic status (SES) or belonging to an ethnic minority often live and work in less favourable environmental conditions, which may result in higher exposure to air, water or soil pollution [3, 4]. Due to lower levels of access to health care, and higher levels of psychological stress and predisposition to diseases, people with a low SES may also be more vulnerable to the adverse health effects of environmental pollution [5, 6]. At the same time, socially disadvantaged groups are systematically underrepresented in biomedical and epidemiological studies that aim to assess the health risks of environmental pollution. This paradox challenges both science and policy. Scientifically, the lower levels of participation of vulnerable social groups introduces a selection bias which undermines the external validity of scientific data. It also hinders subgroup-specific analysis of environmental risk exposure, stratified by income, ethnicity or education, which is one of the major weaknesses in research on environmental health inequalities in the European region according to World Health Organization [7]. This also has consequences for policymaking and society, because by monitoring only middle and higher social classes, specific risk groups remain hidden, impeding targeted policy action and maintaining existing patterns of social exclusion.

The most commonly encountered participation barriers for socially disadvantaged people can be classified into four groups [8–10]. Firstly, there may be psychological or emotional barriers (*feelings*), such as mistrust in science and fear of data abuse and violations of privacy, often the result of social stigma or mistreatment of historically disadvantaged communities. A second group of barriers are more socioeconomic and cognitive in nature (*resources*), such as limited health literacy, which makes the benefits of preventive screening and research of less evident value to those with lower levels of education. A third group comprises cultural barriers (*habits*), which may arise when research designs ignore culturally specific beliefs about illness and health or gender roles. Fourthly, logistical and practical barriers (*obstacles*) may also hinder access to research programmes. These might be rigid exclusion criteria or non-flexible sampling methods.

The challenge of assessing a more diverse study population seems to be particularly difficult in research using human biomonitoring (HBM), a technique used to measure the internal exposure and biological effects of chemical compounds in human tissue or specimens, such as blood, urine or breast milk [11].

Studies comparing the characteristics of participants and nonparticipants in population-based research involving blood donation reveal that socioeconomic factors strongly affect participation [12, 13]. For example, in Spain, individuals with a university education were almost four times more likely to participate in a human biomonitoring study than people with lower levels of education [12]. This may be problematic, since HBM studies in the US [14, 15], Germany [16], Belgium [17] and Spain [18, 19] have shown that socioeconomic factors also influence internal exposure to pollutants. However, these results are not always in line with the traditional environmental justice hypothesis, because social differences have an effect in both directions. Exposure to lead, cadmium, bisphenol A and brominated flame retardants is usually associated with lower SES (measured by educational attainment, income or occupational social class), while body concentrations of mercury, arsenic, and chlorinated compounds such as polychlorinated biphenyls (PCBs) and dichlorodiphenylethylene (DDE) are mostly higher among people with higher SES [14–19]. Besides these larger population-based surveillance studies, HBM data have also been used in (smaller) community-based and advocacy projects that aim to support and empower the voices of specific polluted communities [20]. These projects also emphasise the need for adapted strategies to recruit and retain economically disadvantaged and ethnically diverse participants [21], the challenges of obtaining Institutional Review Board coverage [22], and the need to consider reporting-back protocols in populations with varying levels of literacy [23].

The Flemish Environment and Health Study (FLEHS), a human biomonitoring programme involving newborns, adolescents and adults in Flanders (the northern part of Belgium) [24], faces the same challenges. Participants with lower SES, defined in terms of educational attainment, household income or ethnic background, are systematically underrepresented in the study samples. For example, of the respondents in the combined samples of the FLEHS II studies of mothers and adults (20–40 years of age) in 2008 (*n* = 459), 5.1% were in the low educational attainment group (had not completed secondary education), compared to 14.7% in the general Flemish population (25–34 years of age). 3.9% of respondents had a foreign origin (a parent not born in Belgium), compared to 15% of the general Flemish population. Social scientists from the FLEHS research team have begun to explore this participation bias. To address the issue of social inclusion in the study, we designed, implemented and evaluated a targeted recruitment strategy nested in the FLEHS III mother-newborn study of 2014. This article describes the process and the outcome of this targeted approach, which aimed to increase the participation rate of socially disadvantaged pregnant women and obtain a study sample that was more representative of the social and ethnic diversity of the Flemish population.

## Methods

The aim of the Flemish Environment and Health Study was to provide information on the distribution of internal exposure to environmentally hazardous chemicals in the Flemish population. Flanders is an industrialized region in the north of Belgium with 6 million inhabitants. This region is densely populated and has a dense traffic network. In FLEHS III (2012–2015), participants were recruited from all five Flemish provinces, with the number of participants from each province proportional to the number of inhabitants. A stratified, clustered multi-stage design was used to recruit mothers giving birth in randomly selected maternity hospitals as primary sampling units (PSU). The selected PSUs were located at least 20 km from each other. The standard recruitment strategy within each PSU was to ask midwives to inform pregnant mothers about the study and invite them to participate. Study nurses provided potential participants with detailed information about the study protocol and asked mothers to sign the informed consent form. To account for seasonal variation, recruitment was spread across one full year (November 2013 to November 2014). The inclusion criteria were: (1) residing for at least 5 years in Flanders; (2) giving written informed consent, and (3) being able to fill out an extensive Dutch questionnaire (potentially with the assistance of a family member or an interpreter). All participants donated cord blood and consented to a biopsy from the placenta after the birth of their baby. Optionally, they agreed to donate a hair and/or nail sample. In the week after the delivery, the mothers completed an extensive questionnaire (self-administered) providing information on lifestyle, health status, food consumption, use of tobacco and alcohol, residence history, education and occupation. The study protocol was approved by the ethical committee of the University of Antwerp.

The study goal was to recruit 250 pregnant women. We also aimed to recruit an additional 80 women with a socially more vulnerable position from two of the selected maternity hospitals, one situated in the city of Antwerp (urban region) and one in Heusden-Zolder (rural area in a former coal-mining region). Selection for oversampling was based on three criteria: low educational attainment (mother did not complete secondary education), ethnic background (mother having at least one parent not born in Belgium), and at risk of poverty (having a household income below the national poverty line). In defining ethnic background, we focused on people with Moroccan and Turkish roots, as they constitute the largest ethnic minorities in Belgium [25].

To recruit these hard-to-reach volunteers, we designed a three-track strategy that was implemented in the two maternity hospitals during the entire recruitment period of 13 months.

(I) Modify study procedures – the first step in our strategy was to obtain advice on the ethnic matching and suitability of the vocabulary in the information materials and study procedures. To this end, we conducted eight semi-structured in-depth interviews with organizations and experts in the field of prenatal care, poverty and ethnic cultural minorities. The question guideline for the interviews included four categories: (i) environmental health risk perceptions (how do socially vulnerable pregnant women experience environmental health risks? What are their major environmental concerns?), (ii) habits and beliefs concerning pregnancy and delivery (are there specific cultural habits to be aware of when working with ethnic minority women?), (iii) participation barriers for HBM research (which study procedures may cause barriers for socially vulnerable women?), (iv) opportunities to increase participation (how can we motivate pregnant women? Which elements or messages should be emphasized?). We also organized a focus group with five young mothers from an urban Moroccan and Turkish community. The aim was to test the standard introductory talk about the study protocol and informed consent with the mothers and ask for their feedback. All interviews and focus group were audiotaped in order to extract relevant advice, which was then summarized in separate reports. Finally, we outsourced a thorough screening of our standard information and communication material to a non-profit anti-poverty organization.
(II) Network with community organizations and local professionals – the second step in our strategy was to achieve broader publicity and endorsement of the study and to stimulate word-of-mouth promotion within communities in the catchment areas. To this end, we invested in bilateral consultations with local organizations (e.g. community centres, general practitioners) to personally introduce and advertise the study. We also attempted to encourage and support the midwives in the two selected hospitals in advertising the study to pregnant women with an ethnic background during prenatal consultations.
(III) Implement a personal buddy system for participants – the third and central step in our strategy was to build trust and personal relationships with potential participants by offering them individual support throughout the study process. This was done by buddies – third-party women with the same ethnic background as our target group. We employed three Turkish and Moroccan buddies with a strong social network in the catchment areas close to the collaborating hospital. The buddies were instructed and trained in three consecutive meetings with the research team. They were asked to identify pregnant women eligible for the study, inform them of its aims and encourage them to participate. A brochure summarizing the main messages and expectations was created using culturally appropriate and simply formulated language. This brochure was translated into Arabic, Turkish and French, and included a toll-free phone number for further questions. Buddies gave their personal mobile phone number and e-mail address to interested women to contact them after delivery to arrange a visit in the maternity facility, or at home to assist with filling out or translating the questionnaire. Buddies kept a logbook to document their experiences and record the reactions of potential participants. Halfway through and at the end of the recruitment period, we organized a joint evaluation meeting with the buddies and the research team. We discussed the advantages and disadvantages of the recruitment efforts, the barriers observed and reasons given for study refusal, and the factors associated with willingness to participate.

The results of our targeted recruitment strategy are described in both qualitative and quantitative terms. The qualitative section contains reflections and more formative evaluations of the research team, the buddies and the midwives. Additional input was gathered from a focus group with young mothers of Moroccan and Turkish background, and from consultations with field organizations and poverty experts. The results are structured around the four participation barriers identified. The quantitative results address the summative evaluation and compare the socioeconomic profiles of participants recruited using the targeted strategy and those recruited without this strategy.

## Results

During the design and the implementation of the targeted recruitment strategy, we identified at least four barriers to the participation of disadvantaged people who are at risk of social exclusion from human biomonitoring research.

First barrier: overcoming an intuitive ‘no’. The focus group with Turkish and Moroccan mothers revealed an initial emotional, almost intuitive, barrier to participation in the HBM study. This is related to both the nature of the study (*what* we ask of participants) and the timing of the study (*when* we ask for their participation). The biological samples, particularly the placenta sample, was initially a frightening idea and was perceived to involve giving away personal property. The timing for consent (on the day of delivery) was also concerning because they considered that they would be in ‘an emotional danger zone’. It was suggested that this first psychological barrier might be overcome by the provision of more tangible information about the study and giving candidates more time to decide about participation. If information was provided earlier in the pregnancy, there would be more time to reflect and to consult others in their social environment about study participation and thus prevent people being overcome by this sense of fear when consent is asked for immediately after delivery.

Thus, the primal focus of our recruitment strategy was to invest in information transfer to eligible candidates about the study process in the pre-parturition period. Buddies were employed to inform pregnant women in advance, and maintain contact to assist with filling in the questionnaire after delivery. In the first instance, the buddies were asked to contact pregnant women wherever they thought appropriate. However, we soon realized that pregnant women perceived the HBM study as a genuine medical study (as opposed to a health survey). A personal introductory talk was therefore most appropriate and most effective in a medical setting, that is, the maternity department of the collaborating hospitals. Recruiting pregnant women in public places or at community organization activities was also tried but proved to be less effective because potential candidates could not always be identified (pregnancy not discernible), and the nature of the research was perceived to be too sensitive and personal to address women in a group or in public settings. As an alternative, the buddies were granted access (by the cooperative hospital management) to the prenatal consultation waiting rooms and could approach pregnant Turkish and Moroccan women who they identified as potential candidates. The introductory talk centred on information about the non-invasive and risk-free nature of the biological sampling.

The two most often mentioned reasons for nonparticipation, according to the buddies, were mistrust in the study and lack of husband’s consent. Mistrust was most frequently mentioned as related to anxiety about data abuse. The Turkish buddy mentioned conversations with pregnant women who refused to participate because they believed the placenta sample would be used to clone their baby. The Moroccan buddy observed more religious reservations concerning biological samples. In some Muslim communities, the placenta is perceived as sacred and scientific studies involving tissue donation are believed to be forbidden according to the rules of the Koran (‘haram’). This clearly showed that ethnic minority groups allocate diverse social meanings to and cultural evaluations of placenta donation, and that placental tissue is certainly not universally perceived as biological waste. This has also been documented in other studies [26, 27]. According to the buddies, other women showed an initial interest in participating but expressed the will to discuss this with their husband. When the buddies contacted these women later, they were reluctant to participate because their husband had not consented.

Second barrier: having a perceived benefit. Reassuring the women that study participation would not involve risks seemed to be a first important step, but proved to be inconclusive. The women also needed to be encouraged to complete all elements of the study. The perception of personal utility and benefits of study participation was found to be an important prerequisite, also mentioned in other studies [10, 28]. Typically, the FLEHS research team attempts to underline the benefits of participation by reporting individual results to participants and offering them a summary of the group results. We also attempted to appeal to the social responsibility and scientific inquisitiveness of potential participants by starting our information brochure with the question: ‘*Have you ever wondered whether your child will grow up in a healthy environment? If you have, read on, because we want to answer this question with you’*. However, the pilot testing of this brochure with the target group demonstrated that this message was not convincing. The limited scientific literacy and familiarity with research protocols made it difficult to relate to the scientific and societal value of the study. This was also mentioned in relation to our initial catchphrase in the brochure, intended to articulate the policy relevance of our research: ‘*When health effects are apparent, the government will take action*’. Women living in more deprived conditions could misinterpret this message as entailing the intervention of social services in the domestic arena.

We were advised to frame the utility and benefits of study participation more in terms of ‘personal profit’: firstly, by offering participants more personalized information on how to avoid or protect themselves against exposure to environmentally hazardous chemicals and how to recognize toxic substances in consumer products; and, secondly, by providing more tangible examples of collective policy action being taken in response to the study and emphasizing their valuable contribution to this policy process. We realized, however, that a focus on this information and providing examples required greater familiarity with the study programme and could therefore not effectively be outsourced to the buddies.

**Fig. 1:**
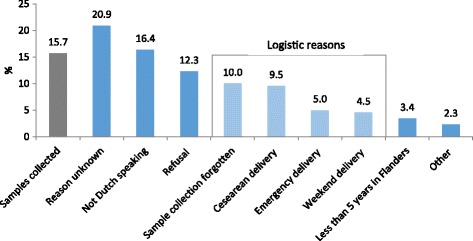
Reasons why samples were not collected in one maternity ward

Third barrier: avoiding research triage. In theory, the midwives had to briefly mention the HBM study and collect samples from all births during the fieldwork period, except from those women who explicitly refused to participate or did not meet the inclusion criteria. In practice, however, we gradually observed multiple logistical restrictions to the sample collection. For example, during low hospital staffing periods (weekends, night shifts) or very busy periods, midwives did not systematically collect samples. Also, in the case of emergency deliveries or caesareans, women were usually not informed about the study and samples were not taken. A detailed monitoring of the sample collection in one maternity ward over 6 months revealed effective sampling in only 16% of deliveries (Fig. 1). In 29% of deliveries, women did not participate in the study for logistical reasons; for example, the midwives forgot to collect the sample, or it was a caesarean, an emergency or a weekend delivery.

Although these obstacles relate to all potential study participants, they particularly affect socially vulnerable populations such as ethnic minorities, who are less confident or less able to communicate with the medical staff in Dutch. Additionally, in relation to these groups, the bias could also be reinforced from the opposite direction: study nurses and midwives may feel less confident in explaining study protocols to non-Dutch-speaking women and assume that they are less interested in participating in biomonitoring research. In clinical trial participation this is called physician triage, a well-known mechanism by which physicians and nurses fail to present the option of participation in a clinical trial because they assume that certain patients would not agree, would not understand the study requirements or would not adhere to protocols [10, 29]. Socially vulnerable women also often leave the hospital within 24 h of delivery and are thus more difficult to reach for the purpose of research studies.

Fourth barrier: filling out the paperwork. Even when samples were taken and consent was given, participants would still be dropped from the study if they did not send back the self-administered questionnaire. Such questionnaires collect data on a broad range of explanatory variables and they can be long and complex, in our case, including questions about diet, the home environment, medical history and work conditions.

The screening of the questionnaire by professionals from a social profit organization indicated that the issue of the simplicity of the questions was a bigger challenge than the language barrier itself, so merely translating the questionnaire was not the best option. The screening of the questionnaire revealed a number of words and phrases that were overly difficult or were confusing to people with weaker literacy skills. At a more fundamental level, a number of questions were identified that would be considered sensitive or offensive, and could be expected to result in socially desirable answers. For example, people living in vulnerable social conditions might attempt to minimize the extent to which they engage in unhealthy behaviour, such as smoking and alcohol consumption, or will not respond to questions concerning sexual health or reproduction. To address these risks, study nurses emphasized the confidentiality of the results to the mothers and actively promoted the support of the buddies or themselves in filling out the questionnaire. The buddies completed several home visits to assist participants to fill out the questionnaire. Although this approach was evaluated positively by the buddies, the Moroccan buddy experienced family tensions in some households when the husband of the participant wanted to monitor the answers given.

After a sample period of 13 months, we recruited 281 mothers across Flanders to FLEHS III: 101 were recruited in the two maternity hospitals where we applied the targeted strategy, and 180 were recruited in four maternity hospitals where the standard recruitment strategy was used. In the previous Flemish HBM study, performed in 2008 (FLEHS II), we recruited 255 mothers in a non-targeted manner.

Table 1 indicates that our targeted approach was most successful in recruiting more participants with a Turkish or Moroccan background. In both maternity hospitals where our strategy was implemented (where Turkish and Moroccan buddies assisted) almost 20.8% of the participants had Turkish or Moroccan roots, compared to 2.2% of the participants in the other four maternity hospitals. Moreover, the percentage of participants with a household income below the national poverty line was 2.4 times higher in the maternity hospitals that used the targeted strategy. These percentages must be interpreted with caution since this was not a standard case-control design. Socioeconomic differences in the samples obtained with and without the targeted recruitment strategy could also be influenced by regional characteristics of the selected maternity hospitals. When we compare the profile of FLEHS III (Column C) with that of FLEHS II where we used the standard strategy in all six maternity facilities (Column D), we see a large increase in the number of participants with a Turkish or Moroccan background, and a moderate increase in participants with a low household income and low educational attainment.

**Table 1:** Socioeconomic profile of participants recruited in FLEHS III (maternity hospitals with and without targeted strategy) and FLEHS II

		FLEHS III 2014			FLEHS II 2008
		(a) Standard strategy in 4 maternity hospitals	(b) Targeted strategy in 2 maternity hospitals	(c) Total in 6 maternity hospitals (a + b)	(d) Total in 6 maternity hospitals
	Recruited participants	*N* = 180	*N* = 101	*N* = 281	*N* = 255
Educational attainment mother	Primary education (less than secondary school)	8.5%	10.4%	9.2%	8.7%
	Secondary education (secondary school)	26.6%	37.5%	30.4%	29.8%
	Tertiary education (college)	65.0%	52.1%	60.4%	61.5%
Ethnic background mother ^a^	Turkish/Moroccan	2.2%	20.8%	8.9%	2.0%
	Belgium	87.3%	69.3%	81.1%	94.1%
	Other	10.0%	9.9%	10.0%	3.9%
Household income ^b^	Below poverty line	10.8%	25.0%	16.4%	12.5%
	Above poverty line	89.2%	75.0%	83.6%	87.5%

^a^Country of birth of parents of mother

^b^Equivalized monthly household income

### Discussion

Socioeconomic participation bias in human biomonitoring studies is a form of environmental injustice within research practice itself: those who are most exposed and most vulnerable are least monitored and least represented in research. In an attempt to address this injustice, we designed and implemented a targeted recruitment strategy to enhance the participation of socially disadvantaged pregnant women. We found that women with a vulnerable social background are not a priori less willing to participate, but they do experience at least four barriers that may hinder their participation. These barriers are related to both psychosocial and situational factors. Lack of trust in the researchers and no perceived study benefits are important personal barriers, while the complex study design, with sampling procedures that were outsourced to external midwives and difficult self-administered questionnaires, were equally important barriers.

To address these barriers, we developed a setup that used personal buddies, which enabled both information transfer about study conditions in the pre-parturition period as well as support and follow-up to assist with the self-administered questionnaires in the post-parturition period. This idea was inspired by case studies which used peer support workers to engage hard-to-reach groups in different fields of healthcare [30]. The results of our study revealed the importance of a direct, person-to-person recruitment method over anonymous and population-based recruitment. This finding is supported by other research showing that random probability sampling (e.g. with computer-generated postcode lists) and indirect and passive methods of recruiting and advertising in public places or through the media are often not effective ways to ensure the ethnic and socioeconomic diversity of the sample [31–34]. Adgate and colleagues [35] found that telephone screening for research participation using a commercial telephone list resulted in the sampling of households with a higher reported income level than the median, thus undersampling the lower socioeconomic groups.

Establishing trust and perceiving the benefits were found to be key to the recruitment process. As other studies have shown, this indicates that people in lower socioeconomic groups perceive biomedical research as more frightening and less beneficial than others [36]. The buddies, therefore, invested in personal introductory talks at the maternity ward to discuss the non-invasive and risk-free nature of the study. This gave pregnant women the opportunity to ask questions, discuss participation with their husbands and make a more informed decision when midwives asked for samples at delivery. Placental perfusion studies involving pregnant women in Finland came to similar conclusions [37, 38]: active communication between participants and recruiters and clear and understandable written information prior to the delivery were crucial for creating trust in the research and obtaining informed consent. Thus, in line with the work of Lind and colleagues [39], we understood recruitment as a matter of involvement rather than persuasion. In addition to trust, perceiving the benefit of study participation was a major facilitator of participation. Schmotzer [10] concluded that patients who perceive they will personally benefit from study participation are the most likely to report they would participate in a research study. We found that the personal benefits of participation in our human biomonitoring was not in the first instance a monetary reward, but rather the opportunity to receive personalized information about environmental health. Providing the women with tangible evidence of their contribution in terms of resulting policy actions was an additional benefit, which has also been mentioned in other studies [34].

Our study also clearly showed the importance of ethnic matching of study design and information materials, and especially of recognizing that husbands act as family gatekeepers. This has also been mentioned in other studies. For example, Dingoyan and colleagues [40] conducted a study with focus groups of Turkish migrants living in Germany on the willingness to participate in health research. The most often stated reasons for lower participation concerned the role of women, lack of knowledge and interest, mistrust and anxiety. Arion et al. [32] also emphasized the importance of husbands acting as family gatekeepers. Out of respect for cultural norms concerning men having authority over family matters, their longitudinal study of mother-child pairs in the US offered candidate mothers the opportunity to have data collectors talk with husbands about potential concerns. The main concerns among husbands were disclosing personal details about the family and that study findings might negatively stereotype Arab Muslims [32]. These examples showed that language is only one barrier that can undermine the communication process alongside other related barriers such as lack of cultural understanding, cultural myths and stereotypes [41].

From the perspective of the researchers, we also found barriers that had to be removed in order to improve participant diversity. Here, flexibility turned out to be key. As Schmotzer [10] stated, researchers have traditionally been rigid in the implementation of protocols. HBM studies with a long-established expertise, such as the Flemish HBM programme, usually benefit from a linear or cyclical research process with fixed routines. However, when recruiting hard-to-reach groups for biomedical research, flexibility and creativity are needed to continually reassess and adjust recruitment methods. This may include, for example, relocating buddies from public places to hospital waiting rooms to approach pregnant women. This approach demands a more organic research process. When efforts to increase sample diversity must fit into an ongoing research project which is based on routines and experience, the linear and organic approaches may conflict, causing friction within the research team. This friction may be further reinforced by the fact that a targeted approach demands extended timeframes and higher staffing and other costs. In our case, the outsourcing of aspects of the fieldwork to buddies and midwives also meant that the researchers lost some control over the recruitment process, which pushed them out of their comfort zone.

If targeted recruitment efforts are to be structurally integrated into future HBM research, it is important to identify solutions that allow flexibility without jeopardizing the scientific quality of the study design. In our research process, we noticed this because our focus on relational ethics – based on empathy and reciprocity – sometimes put pressure on the traditional bioethical principles of autonomy and the privacy of participants. For example, the names and address details of participants, which are usually known only by the study nurses and carefully encrypted, now needed to be shared between study nurses and buddies in order to collect questionnaires and arrange home visits.

This structural integration of a targeted recruitment strategy could be done through the involvement of and deliberation with societal actors early in the research processes, in order to tailor study protocols to specific concerns of the target group. In this respect, investing in more social and cultural diversity in study samples aligns with what could be considered part of the broader EU approach known as Responsible Research and Innovation (RRI). RRI implies that societal actors work together during the entire research process to better align both the process and its outcomes with the values, needs and expectations of society [42].

## Conclusions

By investing in direct, person-to-person contact with trusted buddies and supported by practical advice on cultural and linguistic sensitivity, it is possible to increase the participation rate of socially disadvantaged populations, especially of ethnic minorities, in biomedical research. Improving participant diversity, however, involves more than merely instrumentally tackling access barriers or simplifying information materials. It requires openness and flexibility in the study procedures and, above all, a different mind-set of the researchers involved, which allows study designs to better take into account the experiences and different circumstances of disadvantaged groups.

## Abbreviations

DDE: Dichlorodiphenylethylene
FLEHS: Flemish Environmental and Health Study
HBM: Human biomonitoring
PCBs: Polychlorinated biphenyls
PSU: Primary sampling units
RRI: Responsible research and innovation
SES: Socioeconomic status

## References

[1] Brulle RJ, Pellow DN (2006). Environmental justice: human health and environmental inequalities. Annu Rev Public Health.

[2] Morello-Frosch R, Zuk M, Jerrett M, Shamasunder B, Kyle AD (2011). Understanding the cumulative impacts of inequalities in environmental health: implications for policy. Health Aff.

[3] Mitchell G, Dorling D (2003). An environmental justice analysis of British air quality. Environ Plan A.

[4] Hipp JR, Lakon CM (2010). Social disparities in health: disproportionate toxicity proximity in minority communities over a decade. Health Place.

[5] Wheeler BW, Ben-Shlomo Y (2005). Environmental equity, air quality, socioeconomic status, and respiratory health: a linkage analysis of routine data from the health survey for England. J Epidemiol Community Health.

[6] Gee GC, Payne-Sturges DC (2004). Environmental health disparities: a framework integrating psychosocial and environmental concepts. Environ Health Perspect.

[7] World Health Organization (WHO) (2012). Environmental health inequalities in Europe.

[8] Bonevski B, Randell M, Paul C, Chapman K, Twyman L, Bryant J, Brozek I, Hughes C (2014). Reaching the hard-to-reach: a systematic review of strategies for improving health and medical research with socially disadvantaged groups. BMC Med Res Methodol.

[9] Spears CR, Nolan BV, O'Neill JL, Arcury TA, Grzywacz JG, Feldman SR (2011). Recruiting underserved populations to dermatologic research: a systematic review. Int J Dermatol.

[10] Schmotzer GL (2012). Barriers and facilitators to participation of minorities in clinical trials. Ethnicity & Disease.

[11] Angerer J, Ewers U, Wilhelm M (2007). Human biomonitoring: state of the art. Int J Hyg Environ Health.

[12] Porta M, Gasull M, Puigdomenech E, Rodriguez-Sanz M, Pumarega J, Rebato C, Borrell C (2009). Sociodemographic factors influencing participation in the Barcelona health survey study on serum concentrations of persistent organic pollutants. Chemosphere.

[13] Tjonneland A, Olsen A, Boll K, Stripp C, Christensen J, Engholm G, Overvad K (2007). Study design, exposure variables, and socioeconomic determinants of participation in diet, cancer and health: a population-based prospective cohort study of 57,053 men and women in Denmark. Scand J Public Health.

[14] Tyrrell J, Melzer D, Henley W, Galloway TS, Osborne NJ (2013). Associations between socioeconomic status and environmental toxicant concentrations in adults in the USA: NHANES 2001-2010. Environ Int.

[15] Nelson JW, Scammell MK, Hatch EE, Webster TF (2012). Social disparities in exposures to bisphenol a and polyfluoroalkyl chemicals: a cross-sectional study within NHANES 2003-2006. Environ Health.

[16] Kolossa-Gehring M, Becker K, Conrad A, Ludecke A, Riedel S, Seiwert M, Schulz C, Szewzyk R (2007). German environmental survey for children (GerES IV) - first results. Int J Hyg Environ Health.

[17] Morrens B, Bruckers L, DenHond E, Nelen V, Schoeters G, Baeyens W, Van Larebeke N, Keune H, Bilau M, Loots I (2012). Social distribution of internal exposure to environmental pollution in Flemish adolescents. Int J Hyg Environ Health.

[18] Vrijheid M, Martinez D, Aguilera I, Ballester F, Basterrechea M, Esplugues A, Guxens M, Larranaga M, Lertxundi A, Mendez M (2012). Socioeconomic status and exposure to multiple environmental pollutants during pregnancy: evidence for environmental inequity?. J Epidemiol Community Health.

[19] Gasull M, Pumarega J, Rovira G, López T, Alguacil J, Porta M (2013). Relative effects of educational level and occupational social class on body concentrations of persistent organic pollutants in a representative sample of the general population of Catalonia, Spain. Environ Int.

[20] Washburn R (2013). The social significance of human Biomonitoring. Sociol Compass.

[21] Sexton K, Adgate JL, Church TR, Greaves IA, Ramachandran G, Fredrickson AL, Geisser MS, Ryan AD (2003). Recruitment, retention, and compliance results from a probability study of children's environmental health in economically disadvantaged neighborhoods. Environ Health Perspect.

[22] Brown P, Morello-Frosch R, Brody JG, Altman RG, Rudel RA, Senier L, Pérez C, Simpson R (2010). Institutional review board challenges related to community-based participatory research on human exposure to environmental toxins: a case study. Environ Health.

[23] Morello-Frosch R, Brody JG, Brown P, Altman RG, Rudel R (2009). Toxic ignorance and right-to-know in biomonitoring results communication: a survey of scientists and study participants. Environ Health.

[24] Schoeters G, Den Hond E, Colles A, Loots I, Morrens B, Keune H, Bruckers L, Nawrot T, Sioen I, De Coster S (2012). Concept of the Flemish human biomonitoring programme. Int J Hyg Environ Health.

[25] Phalet K, Deboosere P, Bastiaenssen V (2007). Old and new inequalities in educational attainment - ethnic minorities in the Belgian census 1991-2001. Ethnicities.

[26] Jenkins GL, Sugarman J (2005). The importance of cultural considerations in the promotion of ethical research with human biologic material. J Lab Clin Med.

[27] Yoshizawa RS (2013). Review: public perspectives on the utilization of human placentas in scientific research and medicine. Placenta.

[28] Barnett J, Aguilar S, Brittner M, Bonuck K (2012). Recruiting and retaining low-income, multi-ethnic women into randomized controlled trials: successful strategies and staffing. Contemp Clin Trials.

[29] Barata PC, Gucciardi E, Ahmad F, Stewart DE (2006). Cross-cultural perspectives on research participation and informed consent. Soc Sci Med.

[30] Whittemore R, Rankin SH, Callahan CD, Leder MC, Carroll DL (2000). The peer advisor experience providing social support. Qual Health Res.

[31] Yancey AK, Ortega AN, Kumanyika SK (2006). Effective recruitment and retention of minority research participants. Annu Rev Public Health.

[32] Aroian KJ, Katz A, Kulwicki A (2006). Recruiting and retaining Arab Muslim mothers and children for research. J Nurs Scholarsh.

[33] Gilliss CL, Lee KA, Gutierrez Y, Taylor D, Beyene Y, Neuhaus J, Murrell N (2001). Recruitment and retention of healthy minority women into community-based longitudinal research. J Womens Health Gender-Based Med.

[34] Mohammadi N, Jones T, Evans D (2008). Participant recruitment from minority religious groups: the case of the Islamic population in South Australia. Int Nurs Rev.

[35] Adgate JL, Clayton CA, Quackenboss JJ, Thomas KW, Whitmore RW, Pellizzari ED, Lioy PJ, Shubat P, Stroebel C, Freeman NCG (2000). Measurement of multi-pollutant and multi-pathway exposures in a probability-based sample of children: practical strategies for effective field studies. J Expo Anal Environ Epidemiol.

[36] Wardle J, McCaffery K, Nadel M, Atkin W (2004). Socioeconomic differences in cancer screening participation: comparing cognitive and psychosocial explanations. Soc Sci Med.

[37] Halkoaho A, Pietila AM, Dumez B, Van Damme K, Heinonen S, Vahakangas K (2010). Ethical aspects of human placental perfusion: interview of the mothers donating placenta. Placenta.

[38] Halkoaho A, Pietila AM, Vahakangas K (2011). Ethical aspects in placental perfusion studies: views of the researchers. Placenta.

[39] Lind U, Mose T, Knudsen LE (2007). Participation in environmental health research by placenta donation - a perception study. Environ Health.

[40] Dingoyan D, Schulz H, Mosko M (2012). The willingness to participate in health research studies of individuals with Turkish migration backgrounds: barriers and resources. European Psychiatry.

[41] Hussain-Gambles M, Atkin K, Leese B (2004). Why ethnic minority groups are under-represented in clinical trials: a review of the literature. Health Social Care Community.

[42] Owen R, Macnaghten P, Stilgoe J (2012). Responsible research and innovation. From science in society to science for society, with society. Sci Public Policy.

